# Machine Learning Models for Predicting Adverse Pregnancy Outcomes in Pregnant Women with Systemic Lupus Erythematosus

**DOI:** 10.3390/diagnostics13040612

**Published:** 2023-02-07

**Authors:** Xinyu Hao, Dongying Zheng, Muhanmmad Khan, Lixia Wang, Timo Hämäläinen, Fengyu Cong, Hongming Xu, Kedong Song

**Affiliations:** 1 School of Biomedical Engineering, Faculty of Electronic Information and Electrical Engineering, Dalian University of Technology, Dalian 116024, China; 2 State Key Laboratory of Fine Chemicals, Dalian R&D Center for Stem Cell and Tissue Engineering, Dalian University of Technology, Dalian 116024, China; 3 Department of Obstetrics and Gynecology, Second Affiliated Hospital of Dalian Medical University, Dalian 116027, China; 4 Faculty of Information Technology, University of Jyvaskyla, 40014 Jyvaskyla, Finland; 5 Institute of Zoology, University of the Punjab, Quaid-e-Azam Campus, Lahore 54590, Pakistan; 6 School of Artificial Intelligence, Faculty of Electronic Information and Electrical Engineering, Dalian University of Technology, Dalian 116024, China; 7 Key Laboratory of Integrated Circuit and Biomedical Electronic System, Liaoning Province, Dalian University of Technology, Dalian 116024, China

**Keywords:** prediction, machine learning, systemic lupus erythematosus, SLE, pregnancy, gestation, random forest

## Abstract

Predicting adverse outcomes is essential for pregnant women with systemic lupus erythematosus (SLE) to minimize risks. Applying statistical analysis may be limited for the small sample size of childbearing patients, while the informative medical records could be provided. This study aimed to develop predictive models applying machine learning (ML) techniques to explore more information. We performed a retrospective analysis of 51 pregnant women exhibiting SLE, including 288 variables. After correlation analysis and feature selection, six ML models were applied to the filtered dataset. The efficiency of these overall models was evaluated by the Receiver Operating Characteristic Curve. Meanwhile, real-time models with different timespans based on gestation were also explored. Eighteen variables demonstrated statistical differences between the two groups; more than forty variables were screened out by ML variable selection strategies as contributing predictors, while the overlap of variables were the influential indicators testified by the two selection strategies. The Random Forest (RF) algorithm demonstrated the best discrimination ability under the current dataset for overall predictive models regardless of the data missing rate, while Multi-Layer Perceptron models ranked second. Meanwhile, RF achieved best performance when assessing the real-time predictive accuracy of models. ML models could compensate the limitation of statistical methods when the small sample size problem happens along with numerous variables acquired, while RF classifier performed relatively best when applied to such structured medical records.

## 1. Introduction

Systemic lupus erythematosus (SLE) is a chronic autoimmune inflammatory disease with multi-organ involvement and preferentially affects women of childbearing age [1]. Pregnancy outcomes of SLE patients have been improving owing to advances in medicine; however, lupus pregnancies are still associated with more maternal and fetal complications compared with healthy women. The frequency of lupus flares during pregnancy ranges from 12.7% to 69%; lupus does not spare pregnancy which increases the rate of fetal loss, preterm birth, and small-for-gestational-age (SGA) neonates [2]. Both rheumatologic and obstetric teams need to be alert to adverse pregnancy outcomes.

Early prediction is necessary to improve maternal and neonatal outcomes. The traditional statistical approach to predict categorical disease outcomes involves the use of logistic regression (LR) models. The sample size used for this prediction models is relative to the number of variables, and the ratio between research subjects and variables is widely used as 10 to 1. This minimal sample size criterion has generally been accepted as a methodological quality item in appraising prediction modeling studies; small sample size has frequently been associated with poor predictive performance upon validation [3]. However, considering the very low incidence rate of lupus [4] and even lower rate of childbearing patients with detailed medical records, the small sample size is inevitable and may results in amended or abandoned research [5].

Machine learning (ML) and traditional statistics originate in two different communities but share many similarities, and the former can be considered as a generalization of the latter. Meanwhile, ML shows its own advantages for data analysis. Firstly, there is no strict assumption about the data distribution of variables, which needs extensive data preprocessing. Secondly, although less noise is preferred always, ML can handle noisy data and large variances within the dataset comparatively well. Thirdly, specialized types of ML can be trained on small datasets, especially when the number of features considerably outnumbers the number of observations. Finally, complex ML models can identify complicated, multi-faceted, and non-linear patterns of data efficiently [6]. In recent years, significant progress has been made in applying ML for disease prediction.

**Figure 1:**
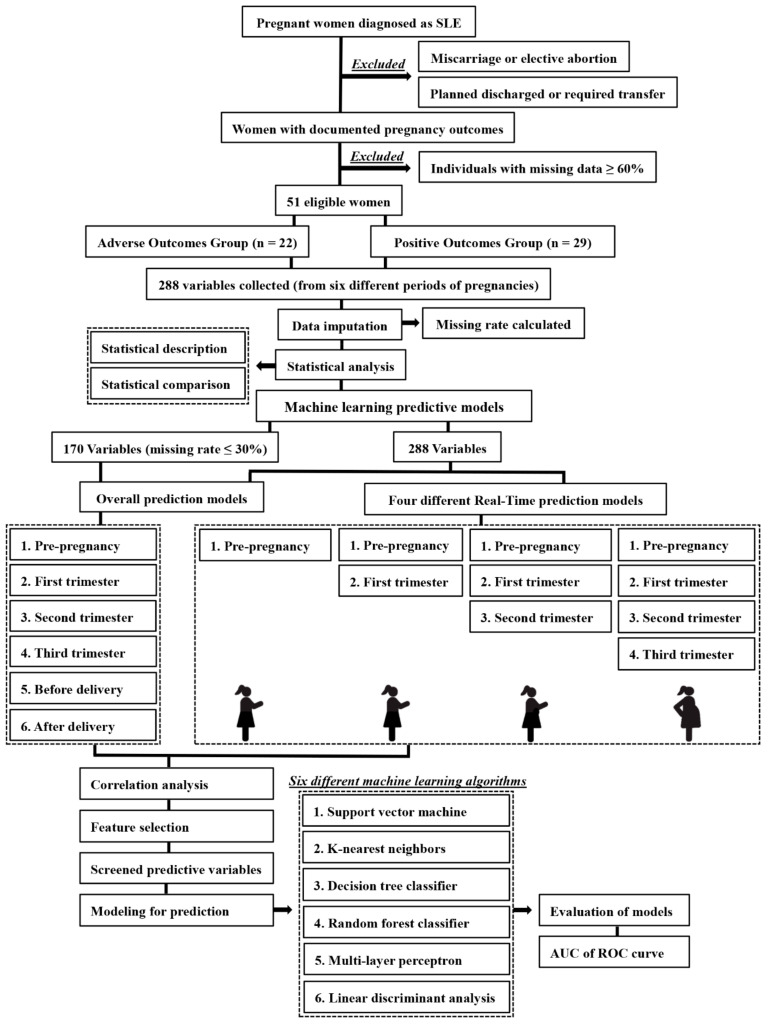
The flow chart of this study design. AUC, area under the curve; ROC, receiver operating characteristic.

In this study, the primary objective was to develop various ML models to predict adverse pregnancy outcomes utilizing a small size dataset with nearly three hundred variables collected before, during, and after gestations and to evaluate the discrimination ability of these models. The second objective was to evaluate the real-time predictive performance of these models, developing the models mentioned above with variables merely from pre-pregnancy care or from pre-pregnancy care associated with prenatal care in different trimesters, in a chronological order, to assess the real-time discriminative ability (the flow chart of this study can be seen in Figure 1).

## 2. Materials and Methods

### 2.1. Study Population

A single-center, retrospective study was conducted. Pregnancy-relevant medical records were reviewed, and eligible women who were diagnosed with SLE before pregnancy with singleton pregnancies were enrolled at Second Affiliated Hospital of Dalian Medical University, from January 2013 to December 2021. All the selected women belong to the Chinese Han population. Participant exclusion criteria: (1) miscarriage or elective abortion; (2) the pregnancy outcomes were unknown, as planned discharged or required transfer; (3) the missing data rate on the analyzed variables was more than 60% [7].

The reason why we excluded patients whose pregnancies ended before 14 weeks was based on the difficulty of identifying the real cause of miscarriage or elective abortion, as the high frequency of miscarriage was contributed to by chromosome errors or endometrial defects [8] instead of SLE, which was impossible to identify in our study; additionally, the real causes of elective abortion are untraceable, for instance an unwanted pregnancy due to drug exposure or the severity of the disease progression.

Upon admission, both clinical and laboratory records were collected, and the records were identified mainly in six different periods, which also varied according to the actual situation. (1) Pre-pregnancy: within six months before pregnancy; (2) first trimester: ≤13 weeks 6 days of gestation; (3) second trimester: ≥14 weeks and ≤27 weeks 6 days of gestation; (4) third trimester: ≥28 weeks of gestation. (5) Before delivery: within 24 h after admission for delivery; (6) after delivery: within three months after delivery. All the specimens were tested at the clinical laboratory of this tertiary care hospital.

Though the new 2019 European League Against Rheumatism (EULAR)/American College of Rheumatology (ACR) SLE classification criteria performed well [9], in this retrospective study, there were no women diagnosed with SLE after the new classification criteria published, and the review of medical records generated before the available date in our hospital or the records from other hospitals is inapplicable because the shared electronic medical record system network is not authorized temporally and spatially. Therefore, SLE was still diagnosed by rheumatologists based on the 1997 ACR criteria for the classification of SLE [10].

Gestational ages were confirmed by ultrasonic examinations before 14 gestational weeks.

### 2.2. Grouping

In order to evaluate the predictive performance of ML models about adverse pregnancy outcomes, we grouped the women as following: (1) Adverse Group (*n* = 22): individuals associated with adverse pregnancy outcomes; (2) Positive Group (*n* = 29): individuals associated with satisfactory pregnancy outcomes (having no adverse outcomes).

Adverse pregnancy outcomes including one or more of the following: (1) fetal death after 13 weeks’ gestation excluded chromosomal abnormalities, anatomical malformation, or congenital infection [11]; (2) early neonatal death (death before 8 days of age) due to complications of prematurity and/or placental insufficiency [12]; (3) preterm delivery at less than 37 weeks due to gestational hypertension, preeclampsia, HELLP syndrome, placental insufficiency, placenta abruption or premature rupture of membranes [12]; (4) SGA neonate (<10th percentile) [12]; (5) fetal distress which was certified by pathological type observed in the cardiotocography [13]; (6) the SLE pregnancy disease activity index (SLEPDAI) was more than 4 [14].

### 2.3. Predictive Variables

Predictive variables include medical history and clinical and laboratory examinations collected before, during, and after pregnancy. The medical records of deliveries and neonates were also collected and assessed. The ultimately enrolled 288 variables were divided into six domains: clinical domain (66 variables), hematologic domain (57 variables), renal domain (56 variables), hepatic domain (30 variables), immunologic domain (75 variables), and thyroid domain (4 variables), listed in Table S1.

Random missing data were an inevitable reality in our retrospective study, which may unnecessarily threaten the validation of results. Therefore, a pre-processing stage is usually required to deal with missing values before any subsequent analysis. K-nearest neighbor intelligent imputation technique can investigate the relationships between attributes and predict both numerical and categorical missing data, so it is an appropriate choice when we have no prior knowledge about the distribution of data. This method is based on the principle that an attribute can be approximated by the values of the “k” attributes that are closest to it [7,15]. After data imputation, a complete dataset was obtained, and the missing data rate was calculated (seen in Table S1).

### 2.4. Statistical Analysis

Descriptive statistics were performed for all variables. Continuous data were presented as medians and interquartile ranges; categorical data were reported in frequencies and percentage. Statistical analyses were performed with the Mann–Whitney U test for continuous or ordinal data and with the chi-squared test or Fisher’s exact test for categorical data between the two groups to determine the differences. *p* value < 0.05 was considered statistically significant.

### 2.5. Correlation Clustering

Correlation of variables were assessed by Spearman’s rank correlation, which is a method of nonparametric statistics. Correlation coefficient ranges from 1 to −1; the closer it is to 1 or −1, then the stronger the correlation between the two variables is. To determine the relationships between all variables, construction of “heat maps” can be led. The display of heat maps solves the problems of pairwise graphic mapping of variables simultaneously and is an illustrative way to assess the presence of dependence [16]. The independent variables screened by correlation analysis would be obtained for following exploration.

### 2.6. Feature Selection

Feature selection is an important data preprocessing step before ML methods are applied to increase prediction accuracy and to decrease computation time consumption. To identify how each variable contributes towards the classification, Decision Tree (DT), an ML method, which would be introduced in detail below, was proposed as the feature selection algorithm. Using each variable to train the DT model, Area Under the Receiver Operator Characteristic Curve (ROC-AUC) was applied to evaluate the predictive accuracy of all the trained DTs; the area under the curve (AUC) was calculated and ranked to reflect the importance of each variables to the prediction task [17,18]. Targeted variables with AUC values more than 0.5 were filtered for subsequent performance.

### 2.7. Model Development

As the purpose of this study was to develop predictive models based on ML algorithms, both the overall models and the real-time models were constructed.

Overall models refer to the models that are constructed by all the variables collected before, during, and after pregnancy, and the predictive ability for adverse outcomes were evaluated. Considering the bias may be produced by the data imputation process, the development of overall models was split into two parts: (1) 288 collected variables were used to construct the overall models regardless of the data missing rate; (2) 170 variables with a missing rate less than 30% were accumulated to develop the overall models. Then, predictive ability of different ML strategies in the two parts were compared, respectively, to comprehend the superior algorithm for modeling and the influence of imputation on modeling.

In addition, real-time models were also developed in order to describe how much time in advance the algorithmic models can achieve the most satisfying discriminative performance for adverse outcomes. We created real-time predictive models as follow: (1) pre-pregnancy models: as the outcomes of 51 participants were known, the variables collected at pre-pregnancy periods were accumulated and utilized to construct the first real-time models, and then the predictive performance for adverse outcomes of these early period models could be figured out based on the AUC values quantitatively; (2) pre-pregnancy + first trimester models: in these models, the modeling variables were collected from pre-pregnancy period to first trimester, as the second real-time models, and the predictive ability of these models were also evaluated; (3) pre-pregnancy + first trimester + second trimester models: with the timespan from pre-pregnancy to the second trimester, the acquired variables were applied to construct the third real-time models; (4) pre-pregnancy + first trimester + second trimester + third trimester models: variables were collected in timespan mentioned above to develop the fourth real-time models.

Data standardization was essential to weaken or even eliminate the disturbance factors of variables with different features and was utilized to solve the problems of comparability between different variables, improving the accuracy of prediction. All the original data were normalized to the same order of magnitude and standardized from 0 to 1 [19]. The description of different ML models is listed below.

Support Vector Machine (SVM), the maximization of separating margin, is a binary linear classifier for classification or regression analysis, creating a decision boundary between two classes that enables the prediction from one or more feature vectors. The model transforms training data into a high-dimensional feature space, separating the decision boundary, known as the hyperplane, with the smallest distance between the hyperplane points and the largest margin between the classes, providing a linear optimal solution [20,21,22].

K-Nearest Neighbor (KNN) is one of the oldest, simplest, and most accurate algorithms for patterns classification and regression models. The core of this classifier depends mainly on measuring the distance or similarity between the tested examples and the training examples. This nonparametric algorithm indicates that there is no fixed number of parameters irrespective of data size and no assumptions about the underlying data distribution. This model could be the best choice for any classification study that involves a little or no prior knowledge about the distribution of the data [23].

The Decision Tree (DT) classifier is a single base classifier consisting of nodes and edges. The building process starts from the root node which is also known as the first split point. This split decides the divisions of the entire dataset on the basis of calculation, and the process continues from top to bottom until partitioning is no longer required. The leaves present at the end of the decision tree represent the last partitions. So far, this system applies to various classification and regression tasks [24].

To overcome the drawbacks of a single base prediction model, the researcher proposed the ensemble learning method, Random Forest (RF), to achieve higher accuracy. The ensemble is composed of multiple decision trees corresponding to various sub-datasets which belong to the same datasets. The algorithm becomes trained with a different subset of features rather than selecting best feature present in the dataset, and this randomness leads to achieve good accuracy. The random forest performs well even though the size of the dataset is very low [24].

The Multi-Layer Perceptron (MLP) is a type of feedforward artificial neural networks with a high degree of connectivity determined by synaptic weights of the network, consisting of three layers: input, hidden, and output layer. In the hidden layer, each artificial neuron contains a nonlinear activation function. Employing the backpropagation algorithm, the training process can be divided into two phases. In forward phase, the synaptic weights are fixed as the signal propagated, while in the backward phase, the error signal propagates backward until it reaches synaptic weight and is adjusted [25].

Linear Discriminant Analysis (LDA) is a multivariate classification technique. Maximizing the ratio of the between-group sum of squares to the within-group sum of squares, this model seeks a linear combination to discriminate multiple measures into two different groups [26]. The decision boundary obtained from the testing sample plays a crucial role in the correct recognition, and linear transformation is performed on data from a higher dimensional space to a lower dimensional space, where the final decision is taken [27].

For all ML models, a ten-fold cross-validation technique was optimized to select the best bias-corrected discriminant model. In this process, the data are divided into ten equal parts. For each iteration, seven parts are used for training, and three parts are used for testing. Ten iterations are performed; each part is used as a testing data in a rotatory manner, and the final performance of models is calculated as the average of all the iterations [28].

### 2.8. Model Testing

As mentioned above, discrimination performance is often visualized using an ROC curve. AUC was assessed to illustrate the classification performance of the models, as well as the sensitivity, specificity, and positive and negative predictive values [29].

All analyses were performed by Python language version 3.6.9, SPSS version 26 (IBM Corp., Armonk, NY, USA), and GraphPad Prism 6.01 (GraphPad Software, San Diego, CA, USA).

### 2.9. Ethics Statement

The requirement for informed consent was waived for this retrospective and observational study. The study protocol was approved by the Ethics Committee of Second Affiliated Hospital of Dalian Medical University (2022-068) and Dalian University of Technology (DUTSCE220416_01). All procedures performed in this study adhered to the ethical standards with the principles of the Declaration of Helsinki. The personal information was shielded before any analysis.

## 3. Results

### 3.1. Characteristics of Pregnant Women with SLE

Fifty-one pregnant women with SLE were included in this retrospective study. The statistical depiction of 288 variables collected before, during, and after pregnancies are listed in Table S1, as well as the calculated missing data rate, and there were 170 variables whose missing rate was less than 30%.

Among the 170 variables, the statistical comparison of medical history and clinical and laboratory examinations between the two groups were conducted, and the variables demonstrating statistical differences were listed in Table 1. There was no statistical difference in age, gravidity, parity, duration of illness, and history of adverse pregnancy between the two groups. Compared with the Positive Group, eighteen variables demonstrated statistical differences in the Adverse Group, and ANA titer collected before delivery indicated significant difference (*p* < 0.001). The gestational age at delivery and birthweight of neonates were significantly different between the two groups as well; meanwhile, there was no difference in the gender of neonates and delivery mode.

### 3.2. Variable Selection Based on Machine Learning Method

**Figure 2:**
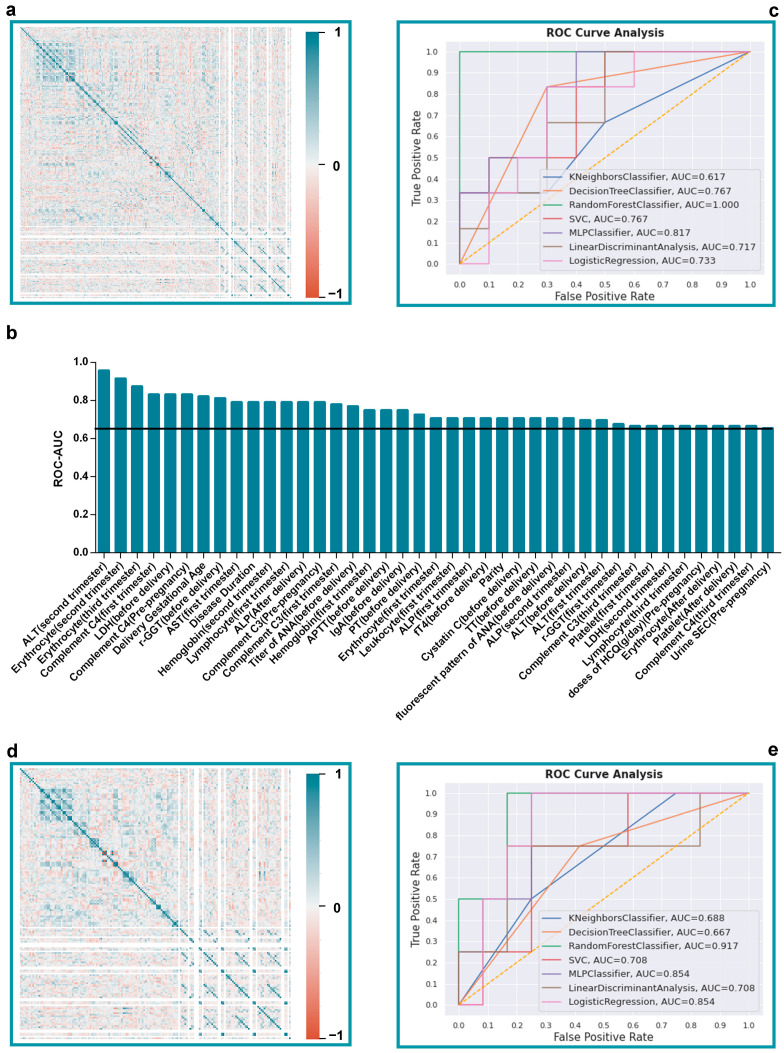
(**a**,**d**) Heat map is an illustrative way of correlation matrix. *x* and *y* axes are variable arrays, and the color of each square corresponds to the calculated correlation coefficients of Spearman correlation test. As the legend on the right indicates, the color of blue indicates a positive correlation, while red indicates a negative correlation. (**a**) 288 variables regardless of missing rate. (**d**) 170 variables with missing rate less than 30%; (**b**) Forty-one variables were selected from 170 variables as important predictors through the AUC values more than 0.65 calculated by DT classifier. The *x*-axis consists of variables, and the *y*-axis represents the AUC values; (**c**,**e**) The ROC-AUC values of six ML predictive models, and logistic regression models were listed as reference. The RF models show the highest AUC values regardless of the missing data rate. (**c**) Predictive models constructed by 288 variables. (**e**) Models developed by 170 variables with low missing rate. SVC, support vector machine.

The first step of data preprocessing before applying ML for prediction models was screening independent variables. As the overall models were constructed in two parts considering the data missing rate, both Figure 2a (288 variables) and Figure 2d (170 variables) show the generated heat maps according to nonparametric Spearman’s rank correlation analysis. The sequence of variables along both X and Y axes are identical to the sequence of variables in Table S1, drawing the illustrative graphs showing correlation relationship between variables.

After removing variables which presented pairwise dependencies, the second step was feature selection relying on the DT classifier. All remaining variables were ranked based on ROC-AUC values, which were listed in Table S2, and the variables with AUC values more than 0.5 would be the targets led to consequent predictive ML models.

As shown in Table 2, among 288 variables, 45 variables with AUC values more than 0.75 were listed, demonstrating their distinctive influence on pregnancy outcomes. Among the 170 variables whose missing rate was less than 30%, the number of selected contributing variables was 41, and the list of variables can be seen in Table 3 and Figure 2b.

However, the obtained variables are quite different from the variables in Table 1 with statistical significance, which indicates the diverse dimensions depicted by two data analysis methods. The overlapped variables in Table 2 and Table 3 with statistical significance were all underlined, and their unique performance recognized by both data analysis methods was determined. Referring to Table 3, ALT collected from the second trimester demonstrated highest level of AUC value, indicating its tight relation with adverse outcome, which presented statistical significance as well. Other variables are Delivery Gestational Age, GGT, Titer of ANA, TT, and Platelet, revealing the importance of hepatic function, autoimmune status, and coagulation function on adverse pregnancy outcomes.

### 3.3. Comparison of Different Machine Learning Models for Overall Prediction

With the expectation of the development of binary classification models for adverse outcome, six ML algorithms were applied to the overall models in two parts, regarding the missing rate, and the ROC graph is shown in Figure 2c,e. Though it is confirmed that the predictive performance of LR can be poor when the prediction model is developed from a dataset with inadequate sample size, the AUC value of LR was still included as reference to witness the predictive accuracy of ML models.

**Figure 3:**
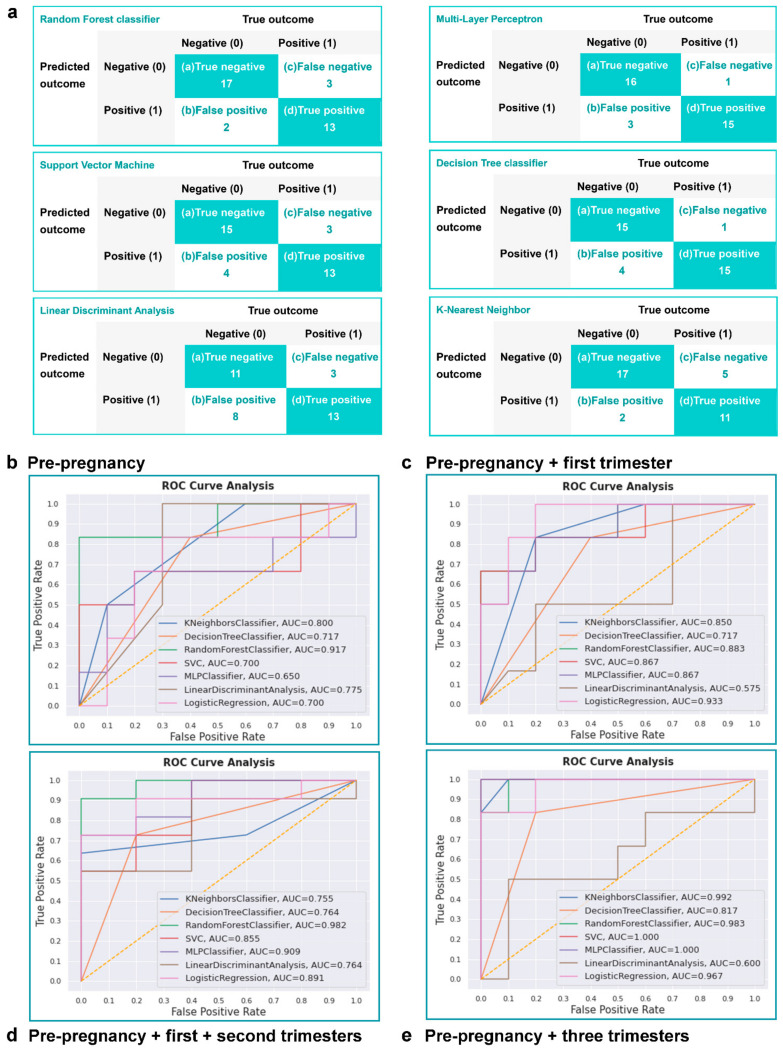
(**a**) The confusion matrixes of six ML models. The results of true positive, true negative, false positive, and false negative of each model are presented in each matrix, and the accuracy, sensitivity, specificity, positive predictive value, and negative predictive value can be calculated based on them; (**b**–**e**) Real-time predictive performance of six ML models and logistic regression in four different timespans testified by ROC-AUC. (**b**) The first timespan is pre-pregnancy period; (**c**) the second timespan is from pre-pregnancy to the first trimester; (**d**) the third timespan is from pre-pregnancy to the second trimester; (**e**) the fourth timespan is from pre-pregnancy to the third trimester.

Referring to the models regardless of missing rate, RF classifier performed best and the AUC of it was 1.000. As shown in Figure 3a, the confusion matrixes of the six classifiers reflect the reliability of decision-making. Noting that 70% of samples were used as training data and that 30% were testing data, the number of samples in confusion matrixes was thirty-five. The RF model demonstrated a sensitivity value of 81.3%, specificity of 89.5%, positive predictive value of 86.7%, and negative predictive value of 85.0%, as shown in Table 4. MLP ranked second with an AUC value of 0.817. KNN (AUC = 0.617) did not show its predictive ability under the current model.

As to the models constructed only by variables with a missing rate less than 30%, which can be seen in Table 5, the RF model was also the superior one, with an AUC value of 0.917, while MLP model ranked second, and the AUC was 0.854. The DT algorithm did not achieve an advantage (AUC = 0.667). It can be determined that removing variables with high missing rate or not, did not tremendously affect the performance ranking of each of the ML strategies.

### 3.4. Comparison of Different Machine Learning Models for Real-Time Prediction

Pregnancy is a complex and dynamic process. To evaluate how much time in advance the six ML models can achieve the most satisfying discriminative performance for adverse outcomes, four different timespans were extracted from the timeline of gestation, and the real-time predictive models applying to current medical records were constructed. As mentioned in the flow chart (Figure 1), we applied six ML models to the screened variables from four timespans and assessed models based on ROC-AUC values. After correlation analysis (four generated heat maps can be seen in Figure S1) and feature selection, contributing variables were ranked in Table S2. The discriminative capability of these real-time models demonstrated by AUC values was illustrated in Table 6 and Figure 3b–e, and LR classification models were utilized as reference once again.

According to the ranking of AUC values in each timespan, the predictive reliability of Random Forest models was testified to identify the advantage of RF algorithm managing the problem of small sample size, in coordination with the superior performance of aforesaid RF model for overall prediction. The ensemble nature of RF classifier helps to outperform individual DT classifier which applies simpler and more straightforward algorithm.

**Figure 4:**
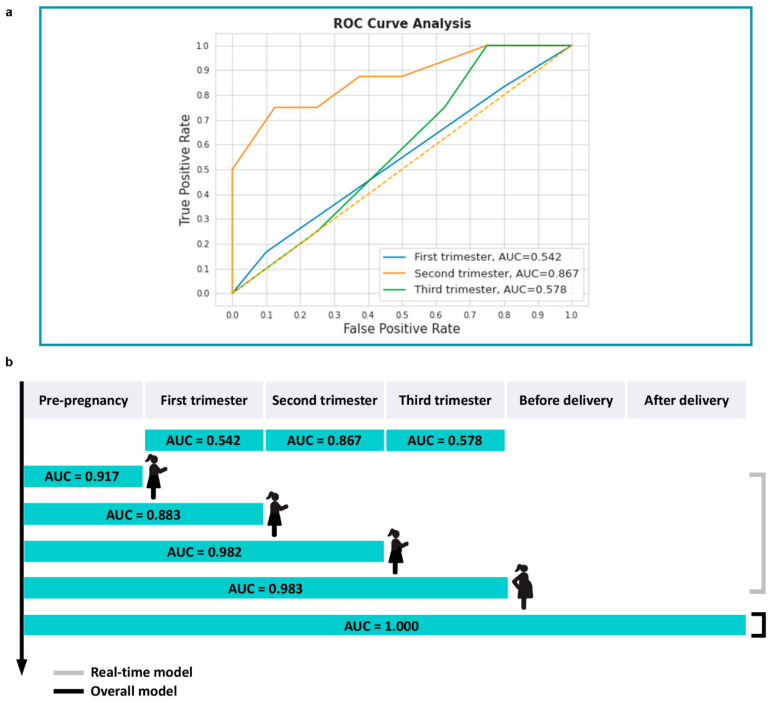
(**a**) AUC values of the RF predictive models constructed by variables merely from first, second, and third trimesters, respectively; (**b**) The AUC values of RF models developed in different gestational trimesters or timespans.

As to the predictive performance of superior RF algorithm, the AUC values demonstrated an interesting variation tendency. Despite the AUC values claimed above, the AUC value of the RF predictive model constructed by variables merely from the first trimester was identified as 0.542; the AUC value from the second trimester was 0.867, and the AUC value from the third trimester was 0.578 (Figure 4a). As shown in Figure 4b, for the current dataset, the predictive ability of adverse outcomes only based on the variables collected before pregnancy was not the strongest (AUC = 0.917), and from the point of clinical view, the risk assessment only implemented before pregnancy is far from adequate; instead of that, close monitoring should be persisted at least until the second trimester. Neither redundant variables accumulated throughout the whole gestation, nor the variables collected from any single trimester will develop the best performed model.

## 4. Discussion

Even if the conception occurs after the period of quiescence, the risk of SLE flare and pregnancy complications can only be minimized and cannot be eliminated. A satisfactory pregnancy management includes the maintenance of low disease activity by rheumatologists, as well as the maternal and fetal monitoring by obstetricians in the whole process of the pregnancy–childbirth–puerperium period. Strengthening the exactness of risk prediction will definitely improve the quality of this cooperative clinical practices and achieve the patient-centered benefits.

Nevertheless, the reality is that the attainable SLE dataset, including the complete tracking records of clinical and laboratory variables during the whole gestation process, might usually come across the problem of insufficient sample size, which means researchers are dealing with a “wide dataset”, where the number of variables exceeds the number of individuals, in contrast to a “long dataset”, where the number of individuals is greater than that of variables. While the classical statistical modeling was designed for the “long dataset”, in the situation of a “wide dataset”, classical statistical inferences become less precise [30]. However, ML prediction models make data-driven classification, which perform the algorithms depends on the pattern of the dataset [6]. After applying six different ML techniques in the current dataset, the first main finding of our study shows that the RF algorithm was testified as a superior model for both overall and real-time adverse outcome predictions, confirmed by ROC-AUC values, a well-established model for discriminative ability of prediction. This technique benefits from the splitting strategy. In the process of creating every decision tree, random variable selection is applied, which makes each decision tree possible to be different from others, improves the diversity of the constructed RF, and guarantees the prediction accuracy [31]. With the advantage of ensemble power, RF can be applicable even in the dataset with highly correlated variables and can achieve good performance in this structural medical dataset stably.

It should be noted that the fundamental purpose of our study is not the competitive comparison between conventional logistic regression analysis and machine learning algorithms for this attempt of binary classification [32,33]; instead, we want to provide ML models as an alternative approach when confronting a dataset with variables outnumbering sample size significantly, such as with rare diseases or genomics data. Clinical practitioners may be more familiar with the thinking of statistical inference and the predicted continuous outcome scores by regression models, while sometimes ML may be helpful to operate the “wide data” problem by finding the generalizable classification patterns automatically.

The second main finding is that the procedure of feature selection is proposed to identify informative variables which may be neglected by traditional statistical analysis. Based on the calculated statistical significance, there are eighteen indicators acquired from different stages of gestation demonstrating statistical differences between the two groups (Table 1); as to the variables selected by feature selection process, even the number of high influential variables with AUC values more than 0.65 is forty-one (Table 3). Compared with the statistical significance based on the assumption that samples are independently and identically distributed, feature selection concentrates on the knowledge of exact distributions of the variables. More and more evidence [34,35,36] has been accumulated that significant variables may not lead to good prediction of outcomes, as more feature selection strategies are applied into variable filtering. Similar to the thinking that ML methods can be alternative approaches for prediction, if prediction is the ultimate goal, we could employ feature selection strategies as alternative approaches for exploring predictive variables and lay aside significance as the only selection criterion. Moreover, ALT collected from the second trimester and GGT, Titer of ANA, TT, and Platelet acquired from different periods of gestation are the predictive variables identified by both the statistical significance and feature selection, indicating the contributing influence of hepatic function, autoimmune status, and coagulation function on adverse pregnancy outcomes.

The third and the last main finding is that risk assessment for adverse pregnancy outcomes neither should be limited to the pre-pregnancy period, nor be delayed until the third trimester, and serious evaluations are suggested to be conducted until the second trimester. The reasons for this emphasis are, as to rheumatologists, the previous studies mainly focus on the importance of disease remission before conception, while as to obstetricians, the former experience may prompt greater focus on the third trimester and delivery which are highly correlated with adverse outcomes. As to the models with AUC values equaling 1, the values do not mean the perfect predictive ability but the over-fitting of the models under current small sample size. Considering this unreliability, the real-time model in the timespan from pre-pregnancy to the second trimester may be the most preferential period to predict adverse outcomes most accurately. Accumulating sufficient but not too redundant information to support clinical decision, this finding may benefit clinical practices but still needs more evidence from similarly designed studies designed.

There are also two limitations in this study. The first limitation is that the missing data rate is relatively high for our retrospective clinical study. There are two main reasons for this. Firstly, in order to reflect what happens in clinical practice, we split the dataset into six different periods instead of taking the whole gestation as the only observation period and designed the four real-time predictive models; hence, the missing date rate in each period was increased inevitably. Secondly, as so far, there is no study providing an evidence-based set of protocols for the frequency of monitoring pregnancies involving SLE [37]. The international or regional consensus on routine maternal and fetal surveillance with practical uniformity and clinical effects is still lacking. Though we employed the KNN imputation method for the missing data, which was testified as the most efficient method in our previous study [38], the results of overall models in two parts testified that removing variables, regardless of their missing rate, did not tremendously affect the ranking of predictive models; while any missing data imputation method is not an ideal circumstance, the development of standard management instructions benefitted the medical work team. Undertaking this task, unified study design concerning different trimesters of gestation with data sharing among multiple centers is quite essential.

In addition, another limitation relates to the preferential strategy of feature selection. Feature selection is the data-fitting pre-processing procedure for ML modeling, aiming for selecting a subset of variables from original dataset based on certain a criterion to develop an efficient classifier with reduced computation consuming. As a diversity of feature selection strategies has been established, different strategies depending on different algorithms and criteria can generate different subsets of variables; therefore, the collections of predictors selected by different strategies for certain predictive model may not overlap completely and may lead model development into uncertainty. Considering the main objectives of this study, we applied DT as the feature selection method, while in the subsequent study, we focus on the performance of different feature selection methods, and the results indicate that the main contributing variables for prediction can be filtered by different selection strategies simultaneously, while the explanation of the selected subsets can only be interpreted by the algorithms themselves, not by clinical knowledge or judgement.

The utilization of ML techniques demonstrated promising potential for exploration of information from “wide data”, where traditional statistics are not applicable. Referring to the long-term tracing medical datasets with small sample sizes and numerous variables, ML can be applied for classification tasks, such as disease diagnosis, evaluation of complication involvement, assessment of adverse outcomes, and prediction of prognosis and late sequelae automatically and efficiently. If so, the real-time classifiers can be embedded in electronic medical record system, and the given alert thresholds will flag the target events in time, triggering the instant surveillance or interventions. However, challenges that match the actual clinical situation, evaluate the actual benefits, and solve the actual problems still need to be concentrated on. Multidisciplinary cooperation from a panel including machine learning experts, traditional statisticians, rheumatologist, and obstetricians in this case manifests the positive energy.

## 5. Conclusions

The machine learning algorithms can be alternatives when the traditional statistical analysis is not applicable, and the utilization of ML models to predict outcomes of pregnancies involving SLE should be encouraged for providing another point of view, as well as a methodology to select influential variables. ALT, GGT, Titer of ANA, TT, and Platelet are the significant predictive variables for adverse outcomes identified by both statistical analysis and feature selection process; The superior discriminative ability of the Random Forest classifier was testified by the results of ROC-AUC when applied to the current dataset regardless of missing rate; the surveillance of pregnancy outcomes should not be limited to the pre-pregnancy period; instead of that, both the rheumatologists and obstetricians should persist the risk assessment based on the accumulated information at least until the second trimester. The future work will focus on the real-time prediction models embedded in the electronic medical records system to alarm the adverse events in real time, which will hopefully benefit SLE women who are pregnant.

## Figures and Tables

**Table 1:** Comparison of clinical and laboratory variables between the two groups.

Variables			Adverse Group (*n* = 22)	Positive Group (*n* = 29)	*p* Value
Age (years)			30.5 (28.8–33.3)	31.0 (29.0–32.5)	0.738
Gravidity			2 (1–2)	2 (1–2)	0.894
Parity			0 (0–0)	0 (0–1)	0.132
Disease duration (years)			8.5 (3–10.3)	8 (4–13.5)	0.696
History of adverse pregnancy	Yes		5 (22.7)	5 (17.2)	0.894
Delivery gestational age			36.9 (34.1–37.9)	39.0 (38.1–39.3)	<0.001
Delivery mode, *n* (%)	Cesarean section		18 (81.8)	19 (65.5)	0.196
	Vaginal delivery		4 (18.2)	10 (34.5)	
Birth weight of neonates			2475 (2065–2907)	3130 (2975–3355)	<0.001
Gender of neonates, *n* (%)	Male		9 (40.9)	18 (62.1)	0.134
Hospitalization after delivery	Yes		4 (18.2)	4 (13.8)	0.970
Doses of hydroxychloroquine (g/day)	≤13 weeks + 6		0.4 (0.2–0.4)	0.2 (0.1–0.4)	0.040
Platelet (×10(9)/L)	14 weeks–27 weeks + 6		168.0 (121.0–202.5)	214.0 (172.0–236.5)	0.005
	28 weeks–31 weeks + 6		154.5 (107.0–192.0)	205.0 (163.0–222.0)	0.025
	Before delivery		148.5 (108.3–219.5)	186.0 (148.5–228.5)	0.047
	After delivery		189.5 (111.0–237.3)	233.0 (206.0–261.0)	0.012
TT (s)	Before delivery		16.4 (15.8–16.9)	15.4 (15.2–16.1)	0.020
Urine casts (/uL)	14 weeks–27 weeks + 6		0.0 (0.0–0.0)	0.1 (0.0–0.3)	0.003
Urine hyaline casts	14 weeks–27 weeks + 6		0.0 (0.0–0.3)	0.3 (0.0–0.5)	0.029
Urine crystals(/uL)	Pre-pregnancy		0.0 (0.0–0.5)	0.1 (0.1–0.7)	0.019
AST (U/L)	14 weeks–27 weeks + 6		22.5 (17.7–24.1)	18.1 (15.0–22.0)	0.026
ALT (U/L)	14 weeks–27 weeks + 6		19.6 (15.6–32.0)	12.7 (9.9–20.1)	0.012
GGT (U/L)	Before delivery		16.5 (11.6–25.7)	11.0 (6.1–16.8)	0.032
	After delivery		17.1 (14.5–40.1)	12.0 (8.6–16.0)	0.004
Complement C4 (g/L)	14 weeks–27 weeks + 6		0.2 (0.2–0.3)	0.2 (0.1–0.2)	0.021
Complement C3 (g/L)	Before delivery		0.9 (0.7–1.0)	1.0 (1.0–1.1)	0.024
ANA titer *n* (%)	Before delivery	1:100	0 (0)	5 (17.2)	<0.001
		1:320	4 (18.2)	15 (51.7)	
		1:1000	13 (59.1)	9 (31.0)	
		1:3200	5 (22.7)	0 (0)	

Data are presented as median value (interquartile range) or number of patients (percentage). Background color of the table distinguished clinical and laboratory variables. TT: Thrombin time; AST: Aspartate Aminotransferase; ALT: Alanine Aminotransferase; GGT: Gamma-Glutamyltransferase; ANA: Anti-nuclear antibody.

**Table 2:** Feature selection of variables with ROC-AUC ≥ 0.75 assessed by DT classifier.

Variables	AUC	Variables	AUC
Delivery Gestational Age	0.817	(14 weeks–27 weeks + 6)	
(Pre-pregnancy)		Cystatin C	0.783
Anti-ds-DNA antibodies	1.000	Lymphocyte	0.767
Creatinine	0.950	(≥28 weeks)	
TSH	0.867	Platelet (28 weeks–32 weeks)	1.000
Complement C3	0.850	Platelet (32 weeks–36 weeks)	1.000
Urea	0.833	ALP (≥28 weeks)	0.950
fT4	0.783	Cystatin C (≥28 weeks)	0.917
GGT	0.783	Leukocyte (≥36 weeks)	0.900
ALP	0.758	IgG (≥28 weeks)	0.900
Urine crystals	0.750	Platelet (≥36 weeks)	0.900
(≤13 weeks + 6)		Complement C4 (≥37 weeks)	0.867
IgG ACA	0.933	Urine SEC (≥28 weeks)	0.808
IgE	0.917	Leukocyte (28 weeks–32 weeks)	0.800
Creatinine	0.900	Uric Acid (≥28 weeks)	0.800
IgA ACA	0.900	GGT (≥28 weeks)	0.783
IgG anti-B2GP1 antibodies	0.850	Urea(≥28 weeks)	0.767
Anti-ds-DNA antibodies	0.850	Urine bacteria(≥28 weeks)	0.750
CRP	0.817	(Before delivery)	
Urea	0.783	Titer of ANA	0.800
(14 weeks–27 weeks + 6)		Cystatin C	0.783
CRP	0.950	(After delivery)	
Urea	0.917	Urea	0.950
ESR	0.908	Uric Acid	0.850
Creatinine	0.867	CRP	0.808
Hemoglobin	0.800	ALP	0.750

The variables underlined are also the variables with statistical significance listed in Table 1. TSH: Thyroid-stimulating hormone; fT4: free thyroxine; ALP: alkaline phosphatase; Ig: Immunoglobulin; ACA: anti-cardiolipin antibodies; CRP: C-reactive protein; ESR: Erythrocyte Sedimentation Rate; SEC: squamous epithelial cells.

**Table 3:** Feature selection of variables with ROC-AUC ≥ 0.65 assessed by DT classifier (only variables with missing rate ≤ 30% were included).

Variables	AUC	Variables	AUC
Delivery Gestational Age	0.823	(14 weeks–27 weeks + 6)	
Disease Duration	0.792	ALP	0.708
Parity	0.708	LDH	0.667
(Pre-pregnancy)		(≥28 weeks)	
Complement C4	0.833	Erythrocyte (28 weeks–32 weeks)	0.875
Complement C3	0.792	Complement C3 (28 weeks–36 weeks + 6)	0.667
Dosage of HCQ	0.667	Lymphocyte (28 weeks–32 weeks)	0.667
Urine SEC	0.656	Complement C4 (28 weeks–36 weeks + 6)	0.667
(≤13 weeks + 6)		(Before delivery)	
Complement C4	0.833	LDH	0.833
AST	0.792	GGT	0.813
Lymphocyte	0.792	Titer of ANA	0.771
Complement C3	0.781	APTT	0.750
Hemoglobin	0.750	IgA	0.750
Erythrocyte	0.708	PT	0.729
Leukocyte	0.708	fT4	0.708
ALP	0.708	Cystatin C	0.708
ALT	0.698	TT	0.708
GGT	0.677	Fluorescent pattern of ANA	0.708
Platelet	0.667	ALT	0.698
(14 weeks–27 weeks + 6)		(After delivery)	
ALT	0.958	ALP	0.792
Erythrocyte	0.917	Erythrocyte	0.667
Hemoglobin	0.792	Platelet	0.667

The variables underlined are also the variables with statistical significance listed in Table 1. HCQ: hydroxychloroquine; LDH: Lactate dehydrogenase; APTT: activated partial thromboplastin time; PT: Prothrombin Time; TT: thrombin time.

**Table 4:** Ranking of different predictive models based on AUC values.

Ranking	All Variables	SEN	SPE	PPV	NPV
1	RF (AUC = 1.000)	0.813	0.895	0.867	0.850
2	MLP (AUC = 0.817)	0.938	0.842	0.833	0.941
3	SVM (AUC = 0.767)	0.813	0.789	0.765	0.833
4	DT (AUC = 0.767)	0.938	0.789	0.789	0.938
5	LDA (AUC = 0.717)	0.813	0.579	0.619	0.786
6	KNN (AUC = 0.617)	0.667	0.895	0.833	0.773
7	LR (AUC = 0.733)	0.688	0.789	0.733	0.750

SEN: sensitivity; SPE: specificity; PPV: positive predictive value; NPV: negative predictive value.

**Table 5:** Ranking of different predictive models based on AUC values (only variables with missing rate ≤ 30% were included).

Ranking	All Variables	SEN	SPE	PPV	NPV
1	RF (AUC = 0.917)	0.889	0.941	0.941	0.889
2	MLP (AUC = 0.854)	0.722	0.588	0.650	0.667
3	SVM (AUC = 0.708)	0.813	0.733	0.765	0.786
4	LDA (AUC = 0.708)	0.500	0.529	0.529	0.500
5	KNN (AUC = 0.688)	0.500	0.471	0.500	0.471
6	DT (AUC = 0.667)	0.611	0.706	0.688	0.632
7	LR (AUC = 0.854)	0.722	0.647	0.684	0.688

**Table 6:** Ranking of real-time predictive models in different timespan based on AUC values.

Ranking	Pre-Pregnancy	Pre-Pregnancy + 1st Trimester	Pre-Pregnancy + 1st + 2nd Trimesters	Pre-Pregnancy + Three Trimesters
1	RF (AUC = 0.917)	RF (AUC = 0.883)	RF (AUC = 0.982)	MLP (AUC = 1.000)
2	KNN (AUC = 0.800)	MLP (AUC = 0.867)	MLP (AUC = 0.909)	SVM (AUC = 1.000)
3	LDA (AUC = 0.775)	SVM (AUC = 0.867)	SVM (AUC = 0.855)	KNN (AUC = 0.992)
4	DT (AUC = 0.717)	KNN (AUC = 0.850)	LDA (AUC = 0.764)	RF (AUC = 0.983)
5	SVM (AUC = 0.700)	DT (AUC = 0.717)	DT (AUC = 0.764)	DT (AUC = 0.817)
6	MLP (AUC = 0.650)	LDA (AUC = 0.575)	KNN (AUC = 0.755)	LDA (AUC = 0.600)
7	LR (AUC = 0.700)	LR (AUC = 0.933)	LR (AUC = 0.891)	LR (AUC = 0.967)

## Data Availability

The datasets analyzed during the current study are available from the corresponding author on reasonable request.

## Supplementary Materials

The following supporting information can be downloaded at: https://www.mdpi.com/article/10.3390/diagnostics13040612/s1. Table S1: Statistical description and missing data rate of 288 variables; Table S2: ROC-AUC calculated by DT for feature selection in overall and real-time periods; Figure S1: Heat maps generated from four timespans.
