# Supplementary material for: Machine Learning Models for Predicting Adverse Pregnancy Outcomes in Pregnant Women with Systemic Lupus Erythematosus
Source: Diagnostics (Basel). 2023 Feb 7;13(4):612. doi: 10.3390/diagnostics13040612 (PMC9955045; doi:10.3390/diagnostics13040612)
Supplement: Supplementary file 1 [file diagnostics-13-00612-s001.zip › Table-S1-Statistical description and missing data rate of 288 variables -marked variables with hight missing rate.pdf]

Table S1. Statistical description and missing data rate of 288 variables.

Variables			Adverse pregnancy outcomes (n=22)	Positive pregnancy outcomes (n=29)	Missing data rate (%)
			**Clinical Domain (66 variables)**		
Age (years)			30.5 (28.8-33.3)	31.0 (29.0-32.5)	0.00
Gravidity			2 (1-2)	2 (1-2)	0.00
Parity			0 (0-0)	0 (0-1)	0.00
Disease duration (years)			8.5 (3-10.3)	8 (4-13.5)	0.00
History of adverse outcomes	Yes		5 (22.7)	5 (17.2)	1.96
Gestational age at Delivery			36.9 (34.1-37.9)	39.0 (38.1-39.3)	0.00
Delivery mode, n (%)	Cesarean section		18 (81.8)	19 (65.5)	0.00
	Vaginal delivery		4 (18.2)	10 (34.5)	
Gender of neonates, n (%)	Male		9 (40.9)	18 (62.1)	0.00
	Female		13 (59.1)	11 (37.9)	
Birth weight of neonates			2475 (2065-2907)	3130 (2975-3355)	0.00
Hospitalization after delivery	Yes		4 (18.2)	4 (13.8)	0.00
Doses of prednisone (mg/day)	Pre-pregnancy		6.3 (5.0-10.0)	7.5 (5.0-10.0)	0.00
	≤ 13 weeks+6		7.5 (5.0-10.0)	7.5 (5.0-10.0)	0.00
	14 weeks-27 weeks+6		8.8 (5.0-10.0)	7.5 (5.0-10.0)	0.00
	≥ 28 weeks		7.5 (5.0-10.0)	7.5 (5.0-10.0)	1.96
Doses of hydroxychloroquine (g/day)	Pre-pregnancy		0.4 (0.2-0.4)	0.2 (0.1-0.4)	0.00
	≤ 13 weeks+6		0.4 (0.2-0.4)	0.2 (0.1-0.4)	0.00
	14 weeks-27 weeks+6		0.4 (0.2-0.4)	0.2 (0.2-0.4)	0.00
	≥ 28 weeks		0.4 (0.2-0.4)	0.2 (0.2-0.4)	1.96
Immunosuppressant	Pre-pregnancy	Yes	2 (9.1)	0 (0)	0.00
	≤ 13 weeks+6	Yes	1 (4.5)	0 (0)	0.00
	14 weeks-27 weeks+6	Yes	3 (13.6)	1 (3.4)	3.92
	≥ 28 weeks	Yes	2 (9.1)	2 (6.9)	1.96
Doses of aspirin (mg/day)	Pre-pregnancy		0 (0.0-0.0)	0 (0.0-0.0)	0.00
	≤ 13 weeks+6		0 (0.0-0.0)	0 (0.0-0.0)	0.00
	14 weeks-27 weeks+6		0 (0.0-0.0)	0 (0.0-25.0)	0.00
	≥ 28 weeks		0 (0.0-0.0)	0 (0.0-25.0)	1.96
Low molecular weight heparin	Pre-pregnancy	Yes	0 (0)	0 (0)	0.00
	≤ 13 weeks+6	Yes	1 (4.5)	2 (6.9)	0.00
	14 weeks-27 weeks+6	Yes	4 (18.2)	5 (17.2)	0.00
	≥ 28 weeks	Yes	4 (18.2)	5 (17.2)	1.96
Painful and swollen joints	Pre-pregnancy	Yes	0 (0)	1 (3.4)	0.00
	≤ 13 weeks+6	Yes	1 (4.5)	0 (0)	0.00

	14 weeks-27 weeks+6	Yes	1 (4.5)	0 (0)	0.00
	≥ 28 weeks	Yes	1 (4.5)	0 (0)	1.96
Rash	Pre-pregnancy	Yes	2 (9.1)	2 (6.9)	0.00
	≤ 13 weeks+6	Yes	2 (9.1)	1 (3.4)	0.00
	14 weeks-27 weeks+6	Yes	2 (9,1)	0 (0)	0.00
	≥ 28 weeks	Yes	3 (13.6)	1 (3.4)	1.96
Alopecia	Pre-pregnancy	Yes	1 (4.5)	2 (6.9)	0.00
	≤ 13 weeks+6	Yes	1 (4.5)	3 (10.3)	0.00
	14 weeks-27 weeks+6	Yes	1 (4.5)	1 (3.4)	0.00
	≥ 28 weeks	Yes	1 (4.5)	1 (3.4)	1.96
Muscle weakness or pain	Pre-pregnancy	Yes	0 (0)	0 (0)	0.00
	≤ 13 weeks+6	Yes	1 (4.5)	1 (3.4)	0.00
	14 weeks-27 weeks+6	Yes	0 (0)	1 (3.4)	0.00
	≥ 28 weeks	Yes	0 (0)	1 (3.4)	1.96
Shortness of breath	Pre-pregnancy	Yes	0 (0)	0 (0)	0.00
	≤ 13 weeks+6	Yes	0 (0)	0 (0)	0.00
	14 weeks-27 weeks+6	Yes	0 (0)	0 (0)	0.00
	≥ 28 weeks	Yes	0 (0)	0 (0)	1.96
Raynaud's phenomenon	Pre-pregnancy	Yes	0 (0)	0 (0)	0.00
	≤ 13 weeks+6	Yes	0 (0)	0 (0)	0.00
	14 weeks-27 weeks+6	Yes	0 (0)	0 (0)	0.00
	≥ 28 weeks	Yes	0 (0)	0 (0)	1.96
Photosensitivity	Pre-pregnancy	Yes	0 (0)	3 (10.3)	0.00
	≤ 13 weeks+6	Yes	0 (0)	2 (6.9)	0.00
	14 weeks-27 weeks+6	Yes	0 (0)	2 (6.9)	0.00
	≥ 28 weeks	Yes	0 (0)	1 (3.4)	1.96
Mucosal ulcers	Pre-pregnancy	Yes	1 (4.5)	1 (3.4)	0.00
	≤ 13 weeks+6	Yes	0 (0)	1 (3.4)	0.00
	14 weeks-27 weeks+6	Yes	0 (0)	1 (3.4)	0.00
	≥ 28 weeks	Yes	0 (0)	1 (3.4)	1.96
Abdominal pain or diarrhea	Pre-pregnancy	Yes	0 (0)	0 (0)	0.00
	≤ 13 weeks+6	Yes	0 (0)	0 (0)	0.00
	14 weeks-27 weeks+6	Yes	0 (0)	0 (0)	0.00
	≥ 28 weeks	Yes	0 (0)	0 (0)	1.96
			**Hematologic Domain (57 variables)**		
Leukocyte (x 10(9)/L)	Pre-pregnancy		6.0 (5.5-7.0)	6.8 (5.1-8.8)	33.33
	≤ 13 weeks+6		5.7 (5.0-8.8)	5.6 (5.2-7.9)	19.61
	14 weeks-27 weeks+6		6.7 (5. 3-10.6)	7.5 (6.5-9.7)	23.53

	28 weeks-31 weeks+6	8.0 (6.6-11.3)	7.8 (6.8-9.9)	29.41
	32 weeks-35 weeks+6	7.6 (6.8-9.5)	7.7 (6.6-10.3)	37.25
	≥ 36 weeks	7.4 (6.5-9.6)	7.7 (6.9-10.1)	41.18
Lymphocyte (x 10(9)/L)	Pre-pregnancy	1.8 (1.5-2.0)	1.8 (1.5-2.1)	31.37
	≤ 13 weeks+6	1.4 (1.1-2.2)	1.6 (1.4-1.8)	19.61
	14 weeks-27 weeks+6	1.4 (0.9-2.1)	1.5 (1.3-1.9)	23.53
	28 weeks-31 weeks+6	1.3 (1.1-2.0)	1.7 (1.3-1.9)	29.41
	32 weeks-35 weeks+6	1.3 (1.2-2.0)	1.6 (1.3-1.9)	37.25
	≥ 36 weeks	1.3 (0.8-1.7)	1.5 (1.2-1.8)	41.18
Neutrophil (x 10(9)/L)	Pre-pregnancy	3.4 (2.9-4.6)	3.4 (3.3-4.4)	31.37
	≤ 13 weeks+6	4.6 (3.4-7.8)	5.2 (3.2-6.9)	19.61
	14 weeks-27 weeks+6	5.1 (4.0-8.4)	5.5 (4.6-7.7)	23.53
	28 weeks-31 weeks+6	6.2 (4.9-8.5)	5.7 (5.0-7.2)	29.41
	32 weeks-35 weeks+6	5.2 (5.0-7.6)	5.9 (4.7-8.0)	37.25
	≥ 36 weeks	6.2 (4.4-7.2)	5.5 (4.6-7.5)	41.18
Platelet (x 10(9)/L)	Pre-pregnancy	218.0 (158.0-329.0)	219.0 (185.0-252.0)	31.37
	≤ 13 weeks+6	176.7 (124.0-234.5)	220.0 (197.0-271.5)	19.61
	14 weeks-27 weeks+6	168.0 (121.0-202.5)	214.0 (172.0-236.5)	23.53
	28 weeks-31 weeks+6	154.5 (107.0-192.0)	205.0 (163.0-222.0)	29.41
	32 weeks-35 weeks+6	158.0 (111.0-227.0)	208.0 (169.0-235.5)	37.25
	≥ 36 weeks	170.0 (116.0-216.0)	198.0 (154.5-209.5)	41.18
	Before delivery	148.5 (108.3-219.5)	186.0 (148.5-228.5)	0.00
	After delivery	189.5 (111.0-237.3)	233.0 (206.0-261.0)	19.61
Erythrocyte (x 10(12)/L)	Pre-pregnancy	4.3 (4.3-4.6)	4.3 (4.2-4.5)	31.37
	≤ 13 weeks+6	4.4 (4.0-4.8)	4.2 (3.9-4.6)	19.61
	14 weeks-27 weeks+6	4.2 (3.7-4.4)	4.0 (3.8-4.3)	23.53
	28 weeks-31 weeks+6	4.0 (3.8-4.3)	3.8 (3.7-4.2)	29.41
	32 weeks-35 weeks+6	3.8 (3.6-3.9)	4.0 (3.7-4.2)	37.25
	≥ 36 weeks	3.8 (3.5-4.2)	3.9 (3.8-4.1)	41.18
	Before delivery	3.9 (3.6-4.3)	3.9 (3.7-4.2)	0.00
	After delivery	4.7 (4.4-4.9)	4.5 (4.3-4.7)	19.61
Hematocrit (%)	Pre-pregnancy	38.6 (35.4-41.7)	38.9 (37.8-39.6)	31.37
	≤ 13 weeks+6	38.3 (35.6-41.1)	37.3 (35.7-39.0)	19.61
	14 weeks-27 weeks+6	37.3 (31.8-39.5)	36.0 (34.4-37.7)	23.53
	28 weeks-31 weeks+6	36.5 (33.0-37.7)	35.9 (33.6-38.1)	29.41
	32 weeks-35 weeks+6	35.1 (32.3-35.6)	34.8 (34.2-36.3)	37.25
	≥ 36 weeks	34.7 (31.6-39.0)	35.8 (34.5-37.4)	41.18
	Before delivery	35.3 (31.5-40.3)	36.7 (35.3-38.9)	0.00
	After delivery	41.9 (39.1-43.9)	40.2 (38.6-41.7)	19.61
Hemoglobin (g/L)	Pre-pregnancy	129.5 (116.3-139.0)	132.0 (127.0-134.5)	31.37
	≤ 13 weeks+6	127.0 (119.0-137.5)	127.0 (123.0-133.5)	19.61
	14 weeks-27 weeks+6	126.5 (106.3-135.0)	122.0 (117.0-128.0)	23.53

	28 weeks-31 weeks+6		124.5 (110.0-128.5)	119.0 (112.5-126.0)	29.41
	32 weeks-35 weeks+6		117.0 (108.5-122.5)	117.2 (115.0-122.5)	37.25
	≥ 36 weeks		115.0 (106.0-133.0)	120.0 (113.0-123.0)	41.18
	Before delivery		117.0 (98.8-134.5)	125.0 (114.5-129.0)	0.00
	After delivery		136.0 (126.3-143.5)	132.0 (124.5-136.5)	19.61
PT (s)	Before delivery		12.1 (11.7-12.4)	11.8 (11.7-12.5)	1.96
PT-act (%)	Before delivery		119.5 (107.0-130.3)	125.0 (111.0-136.0)	1.96
PT-R	Before delivery		0.9 (0.9-1.0)	0.9 (0.9-1.0)	1.96
INR	Before delivery		0.9 (0.9-1.0)	0.9 (0.9-1.0)	1.96
Fbg (g/L)	Before delivery		4.3 (4.0-4.8)	4.3 (3.8-4.8)	1.96
APTT (s)	Before delivery		34.6 (31.5-39.7)	32.3 (30.2-33.9)	1.96
TT (s)	Before delivery		16.4 (15.8-16.9)	15.4 (15.2-16.1)	1.96
			**Renal Domain (56 variables)**		
Urine protein (g/L), n (%)	Pre-pregnancy	negative	5 (22.7)	12 (41.4)	41.18
		(+-)	3 (13.6)	6 (20.7)	
		(+)	14 (63.6)	9 (31.0)	
		(++)	0 (0)	2 (6.9)	
	≤ 13 weeks+6	negative	9 (40.9)	10 (34.5)	19.61
		(+-)	1 (4.5)	1 (3.4)	
		(+)	11 (50.0)	13 (44.8)	
		(++)	1 (4.5)	5 (17.2)	
	14 weeks-27 weeks+6	negative	9 (40.9)	12 (41.4)	15.69
		(+-)	3 (13.6)	5 (17.2)	
		(+)	9 (40.9)	11 (37.9)	
		(++)	1 (4.5)	1 (3.4)	
	≥ 28 weeks	negative	13 (59.1)	14 (48.3)	31.37
		(+-)	9 (40.9)	11 (37.9)	
		(+)	0 (0)	4 (13.8)	
		(++)	0 (0)	0 (0)	
Urine erythrocyte counting (/HP)	Pre-pregnancy		0.0 (0.0-1.0)	0.0 (0.0-0.0)	41.18
	≤ 13 weeks+6		0.0 (0.0-1.0)	0.0 (0.0-0.0)	19.61
	14 weeks-27 weeks+6		0.0 (0.0-0.0)	0.0 (0.0-0.0)	15.69
	≥ 28 weeks		0.0 (0.0-0.2)	0.0 (0.0-0.0)	31.37
Urinary casts (/μL)	Pre-pregnancy		0.0 (0.0-0.2)	0.0 (0.0-0.3)	29.41
	≤ 13 weeks+6		0.0 (0.0-0.2)	0.2 (0.0-0.3)	35.29
	14 weeks-27 weeks+6		0.0 (0.0-0.0)	0.1 (0.0-0.3)	19.61
	≥ 28 weeks		0.0 (0.0-0.7)	0.0 (0.0-0.5)	3.92
Urine hyaline casts (/μL)	Pre-pregnancy		0.0 (0.0-0.4)	0.1 (0.0-0.6)	29.41
	≤ 13 weeks+6		0.3 (0.0-0.5)	0.3 (0.0-0.6)	35.29
	14 weeks-27 weeks+6		0.0 (0.0-0.3)	0.3 (0.0-0.5)	19.61
	≥ 28 weeks		0.0 (0.0-0.6)	0.0 (0.0-0.6)	3.92

Urine squamous epithelial cells (/μL)	Pre-pregnancy	1.4 (0.0-25.6)	5.7 (3.6-22.4)	29.41
	≤ 13 weeks+6	5.2 (0.0-26.8)	6.6 (0.8-16.7)	35.29
	14 weeks-27 weeks+6	2.8 (0.0-8.8)	12.9 (0.0-19.7)	19.61
	≥ 28 weeks	2.3 (0.0-13.8)	4.0 (0.7-7.9)	3.92
Urine bacteria (/μL)	Pre-pregnancy	7.3 (0.0-359.9)	107.0 (27.3-672.9)	29.41
	≤ 13 weeks+6	27.5 (0.0-378.9)	113.1 (66.9-1248.9)	35.29
	14 weeks-27 weeks+6	30.4 (0.0-371.0)	110.3 (0.0-221.0)	19.61
	≥ 28 weeks	104.3 (0.0-259.9)	64.0 (34.0-211.1)	3.92
Urine yeast (/μL)	Pre-pregnancy	0.0 (0.0-0.0)	0.0 (0.0-0.0)	29.41
	≤ 13 weeks+6	0.0 (0.0-0.0)	0.0 (0.0-0.0)	35.29
	14 weeks-27 weeks+6	0.0 (0.0-0.0)	0.0 (0.0-0.0)	19.61
	≥ 28 weeks	0.0 (0.0-0.0)	0.0 (0.0-0.0)	3.92
Urine crystals (/μL)	Pre-pregnancy	0.0 (0.0-0.5)	0.1 (0.1-0.7)	29.41
	≤ 13 weeks+6	0 (0-0.6)	0.4 (0.0-1.3)	35.29
	14 weeks-27 weeks+6	0 (0-0.1)	0.1 (0-0.2)	19.61
	≥ 28 weeks	0 (0-0.7)	0 (0-0.5)	3.92
Creatinine (μmol/L)	Pre-pregnancy	61.0 (56.3-61.8)	54.0 (46.9-61.2)	45.10
	≤ 13 weeks+6	49.3 (47.0-59.6)	51.0 (41.0-57.8)	33.33
	14 weeks-27 weeks+6	49.7 (48.5-53.5)	42.2 (37.8-52.7)	35.29
	≥ 28 weeks	50.2 (37.0-52.3)	43.4 (39.9-48.9)	45.10
	Before delivery	50.6 (43.6-62.5)	48.4 (43.0-50.6)	7.84
	After delivery	60.8 (54.3-64.7)	52.6 (48.6-59.0)	35.29
Cystatin C (mg/L)	Pre-pregnancy	0.9 (0.8-1.0)	0.7 (0.7-0.9)	45.10
	≤ 13 weeks+6	0.8 (0.7-1.1)	0.7 (0.7-0.9)	33.33
	14 weeks-27 weeks+6	0.9 (0.9-1.1)	0.8 (0.6-1.1)	35.29
	≥ 28 weeks	1.0 (0.8-1.1)	0.9 (0.7-1.0)	45.10
	Before delivery	1.3 (1.1-1.6)	1.2 (1.1-1.6)	9.80
	After delivery	0.9 (0.9-1.1)	0.9 (0.8-1.0)	37.25
Urea (mmol/L)	Pre-pregnancy	4.1 (3.8-4.9)	4.1 (3.1-4.7)	45.10
	≤ 13 weeks+6	3.0 (2.4-3.9)	3.2 (2.4-4.0)	33.33
	14 weeks-27 weeks+6	3.2 (2.5-3.8)	2.6 (2.2-3.2)	35.29
	≥ 28 weeks	3.1 (2.6-3.2)	2.6 (2.2-3.5)	45.10
	Before delivery	3.8 (2.6-5.0)	3.7 (2.7-4.2)	7.84
	After delivery	5.2 (3.8-6.4)	4.7 (3.6-5.9)	35.29
Uric acid (μmol/L)	Pre-pregnancy	310.3 (308.8-334.6)	284.9 (236.5-331.5)	45.10
	≤ 13 weeks+6	284.3 (244.2-335.4)	224.5 (200.0-277.5)	33.33
	14 weeks-27 weeks+6	286.9 (241.6-340.2)	230.9 (201.6-268.6)	35.29
	≥ 28 weeks	268.1 (244.0-321.2)	269.6 (230.1-307.4)	45.10
	Before delivery	333.7 (277.5-399.4)	324.1 (295.3-356.2)	9.80
	After delivery	362.6 (281.3-384.7)	354.8 (272.2-385.1)	35.29
		**Hepatic Domain (30 variables)**		
ALT (U/L)	Pre-pregnancy	16.3 (11.0-22.9)	16.1 (13.0-21.5)	33.33
	≤ 13 weeks+6	19.0 (14.8-35.0)	16.0 (10.9-22.7)	15.69

	14 weeks-27 weeks+6		19.6 (15.6-32.0)	12.7 (9.9-20.1)	21.57
	≥ 28 weeks		19.1 (15.5-27.0)	17.6 (12.9-22.0)	37.25
	Before delivery		15.5 (10.1-22.0)	12.4 (10.0-15.5)	7.84
	After delivery		23.1 (15.9-31.0)	16.0 (11.5-29.0)	23.53
AST (U/L)	Pre-pregnancy		20.4 (17.8-22.1)	17.3 (15.9-20.5)	33.33
	≤ 13 weeks+6		20.5 (16.2-25.2)	18.4 (15.3-23.7)	15.69
	14 weeks-27 weeks+6		22.5 (17.7-24.1)	18.1 (15.0-22.0)	21.57
	≥ 28 weeks		20.8 (18.6-22.7)	20.0 (16.8-22.6)	37.25
	Before delivery		19.7 (17.0-24.1)	21.0 (16.2-23.2)	7.84
	After delivery		19.9 (16.6-25.9)	18.0 (15.5-21.1)	23.53
ALP (U/L)	Pre-pregnancy		45.2(37.4-60.0)	43.0 (35.0-54.4)	33.33
	≤ 13 weeks+6		45.6 (39.5-62.0)	47.6 (40.7-61.0)	15.69
	14 weeks-27 weeks+6		46.1 (42.2-68.2)	47.0 (37.0-90.5)	21.57
	≥ 28 weeks		62.3 (39.8-78.5)	56.9 (43.9-66.9)	39.22
	Before delivery		129.1 (99.0-171.3)	138.0 (111.7-194.5)	7.84
	After delivery		67.0 (58.9-73.6)	76.0 (58.9-82.0)	23.53
GGT (U/L)	Pre-pregnancy		16.7 (9.6-28.3)	13.0 (9.0-15.6)	33.33
	≤ 13 weeks+6		19.1 (12.5-30.6)	16.0 (7.5-24.2)	15.69
	14 weeks-27 weeks+6		25.0 (12.0-32.9)	10.4 (7.5-19.7)	21.57
	≥ 28 weeks		22.3 (6.5-27.0)	15.3 (9.8-19.1)	37.25
	Before delivery		16.5 (11.6-25.7)	11.0 (6.1-16.8)	7.84
	After delivery		17.1 (14.5-40.1)	12.0 (8.6-16.0)	23.53
LDH (U/L)	Pre-pregnancy		192.4 (180.6-213.0)	182.0 (176.0-226.9)	33.33
	≤ 13 weeks+6		195.8 (177.0-204.5)	189.0 (170.0-199.7)	15.69
	14 weeks-27 weeks+6		194.9 (183.7-225.0)	188.6 (171.0-215.5)	21.57
	≥ 28 weeks		204.1 (174.7-213.8)	184.0 (160.4-198.0)	39.22
	Before delivery		215.3 (195.8-260.4)	211.0 (191.4-232.5)	9.80
	After delivery		206.1 (187.8-220.0)	185.0 (177.2-224.0)	25.49
**Immunologic Domain (75 variables)**					
ANA titer, n (%)	Before delivery	1:100	0 (0)	5 (17.2)	25.49
		1:320	4 (18.2)	15 (51.7)	
		1:1000	13 (59.1)	9 (31.0)	
		1:3200	5 (22.7)	0 (0)	
Fluorescent pattern of ANA, n (%)	Before delivery	Speckled	13 (59.1)	15 (51.7)	25.49
		Homogeneous	4 (18.2)	1 (3.4)	
		Cytoplasmic	0 (0)	0 (0)	
		Homogeneous+Cytoplasmic	0 (0)	2 (6.9)	
		Speckled+Cytoplasmic	4 (18.2)	5 (17.2)	
		Speckled+Homogeneous	1 (4.5)	5 (17.2)	
		Nucleolar	0 (0)	1 (3.4)	
Complement C3 (g/L)	Pre-pregnancy		0.9 (0.9-1.0)	0.9 (0.8-1.0)	29.41

	≤ 13 weeks+6	0.9 (0.9-1.0)	1.0 (0.9-1.1)	27.45
	14 weeks-27 weeks+6	1.0 (0.9-1.1)	1.0 (0.9-1.1)	23.53
	28 weeks-36 weeks+6	1.0 (0.9-1.1)	1.1 (1.0-1.3)	23.53
	≥ 37 weeks	1.0 (0.9-1.1)	1.0 (1.0-1.1)	43.14
	Before delivery	0.9 (0.7-1.0)	1.0 (1.0-1.1)	7.84
	After delivery	1.0 (0.8-1.1)	1.1 (0.9-1.1)	17.65
Complement C4 (g/L)	Pre-pregnancy	0.2 (0.1-0.2)	0.2 (0.1-0.2)	29.41
	≤ 13 weeks+6	0.2 (0.2-0.2)	0.2 (0.1-0.2)	27.45
	14 weeks-27 weeks+6	0.2 (0.2-0.3)	0.2 (0.1-0.2)	23.53
	28 weeks-36 weeks+6	0.2 (0.1-0.2)	0.2 (0.1-0.2)	23.53
	≥ 37 weeks	0.2 (0.1-0.2)	0.2 (0.1-0.2)	43.14
	Before delivery	0.2 (0.1-0.2)	0.2 (0.1-0.2)	7.84
	After delivery	0.2 (0.2-0.3)	0.2 (0.2-0.2)	17.65
CRP (mg/L)	≤ 13 weeks+6	3.1 (3.0-3.2)	3.3 (3.1-3.6)	49.02
	14 weeks-27 weeks+6	3.1 (3.0-3.5)	3.5 (3.1-4.9)	52.94
	Before delivery	5.2 (3.0-12.4)	4.3 (3.3-31.9)	27.45
	After delivery	3.2 (3.1-13.5)	3.1 (3.0-4.3)	52.94
ESR (mm/h)	Pre-pregnancy	7.0 (4.8-26.0)	9.0 (5.0-16.5)	35.29
	≤ 13 weeks+6	18.0 (13.5-22.0)	10.0 (6.0-17.0)	41.18
	14 weeks-27 weeks+6	21.0 (9.0-23.3)	15.0 (8.0-19.5)	41.51
	Before delivery	16.0 (8.0-28.8)	9.0 (4.0-22.5)	33.33
	After delivery	8.0 (6.0-17.0)	17.0 (5.0-22.0)	35.29
PCT (ng/mL)	Before delivery	0.04 (0.04-0.06)	0.04 (0.04-0.05)	62.75
Ig A (g/L)	Pre-pregnancy	2.8 (2.2-3.2)	2.7 (2.2-4.2)	52.94
	≤ 13 weeks+6	2.1 (1.4-3.0)	2.9 (2.1-3.6)	52.94
	14 weeks-27 weeks+6	2.2 (2.0-2.7)	2.0 (1.6-2.5)	50.98
	≥ 28 weeks	2.3 (1.8-3.1)	2.1 (1.4-2.4)	37.25
	Before delivery	1.8 (1.3-2.6)	2.2 (1.6-2.7)	17.65
	After delivery	2.7 (2.2-3.0)	2.7 (2.0-3.5)	47.06
Ig E (IU/mL)	Pre-pregnancy	37.5 (4.7-120.0)	76.1 (20.1-219.0)	52.94
	≤ 13 weeks+6	60.3 (4.7-133.0)	59.7 (18.7-208.0)	52.94
	14 weeks-27 weeks+6	75.5 (4.7-117.3)	46.4 (15.3-197.5)	50.98
	≥ 28 weeks	93.2 (12.0-289.0)	38.8 (15.4-211.0)	35.29
	Before delivery	64.6 (10.1-130.8)	37.9 (15.9-145.0)	17.65
	After delivery	121.0 (10.3-214.8)	35.2 (17.3-118.1)	47.06
Ig G (g/L)	Pre-pregnancy	14.9 (13.8-16.4)	16.3 (12.2-17.5)	52.94
	≤ 13 weeks+6	12.1 (10.3-14.0)	13.4 (12.7-14.9)	52.94
	14 weeks-27 weeks+6	12.3 (11.5-14.7)	12.7 (10.0-13.5)	50.98
	≥ 28 weeks	12.4 (12.0-16.2)	11.5 (9.7-13.8)	35.29
	Before delivery	11.4 (9.0-14.5)	9.3 (8.6-11.5)	17.65
	After delivery	15.2 (13.2-16.5)	14.5 (12.3-16.9)	47.06

Ig M (g/L)	Pre-pregnancy		0.9 (0.8-1.3)	1.1 (0.6-1.4)	52.94
	≤ 13 weeks+6		0.7 (0.5-1.1)	1.1 (0.6-1.6)	52.94
	14 weeks-27 weeks+6		0.7 (0.5-1.0)	1.0 (0.5-1.1)	50.98
	≥ 28 weeks		0.9 (0.7-1.0)	0.9 (0.5-1.3)	35.29
	Before delivery		0.7 (0.4-0.9)	0.8 (0.5-1.0)	17.65
	After delivery		0.8 (0.7-0.9)	0.9 (0.7-1.3)	47.06
IgA anti-B2GP1 antibodies (RU/mL)	≤ 13 weeks+6		1.6 (1.0-6.2)	9.2 (2.8-13.9)	56.86
	Before delivery		0.0 (0.0-4.7)	0.5 (0.0-5.2)	52.94
IgG anti-B2GP1 antibodies (RU/mL)	≤ 13 weeks+6		2.4 (1.1-3.7)	2.7 (1.1-8.8)	60.78
	Before delivery		1.6 (0.4-2.6)	1.0 (0.7-4.0)	54.90
IgM anti-B2GP1 antibodies (RU/mL)	≤ 13 weeks+6		2.5 (0.4-9.2)	1.9 (0.4-3.5)	60.78
	Before delivery		2.9 (0.1-19.3)	0.8 (0.0-4.0)	54.90
IgA ACA (RU/mL)	≤ 13 weeks+6		1.7 (1.4-2.7)	1.1 (0.0-3.7)	52.94
	Before delivery		1.3 (0.0-1.8)	1.4 (0.8-3.5)	52.94
IgG ACA (RU/mL)	≤ 13 weeks+6		1.3 (0.0-7.6)	0.0 (0.0-2.6)	39.22
	Before delivery		1.0 (0.0-4.8)	0.0 (0.0-2.6)	33.33
IgM ACA (RU/mL)	≤ 13 weeks+6		2.4 (0.5-3.0)	0.4 (0.0-1.4)	39.22
	Before delivery		0.2 (0.0-1.8)	0.0 (0.0-0.7)	33.33
Anti-ds-DNA antibodies (IU/mL)	Pre-pregnancy		42.8 (7.2-78.5)	81.5 (34.3-194.5)	31.37
	≤13 weeks+6		54.2 (20.2-208.3)	122.5 (20.8-145.0)	33.33
	14 weeks-27 weeks+6		39.2 (12.8-217.1)	67.5 (26.9-153.0)	43.14
	≥ 28 weeks		36.0 (6.7-255.7)	69.0 (21.0-175.2)	50.98
	Before delivery		57.3 (15.7-186.9)	46.6 (19.8-100.0)	5.88
	After delivery		31.7 (3.8-136.2)	80.1 (23.5-156.7)	39.22
Anti-C1q antibodies (RU/mL)	Before delivery		2.2 (1.3-9.7)	1.1 (0.0-5.8)	49.02
Anti-nucleosome antibodies (RU/mL)	Before delivery		0.4 (0.0-6.0)	0.8 (0.0-5.0)	29.41
Anti-ribosomal P protein antibodies (RU/mL)	Before delivery		1.8 (1.1-7.7)	1.4 (0.4-50.3)	47.06
Anti-histone antibodies, n (%)	Before delivery	negative	21 (95.5)	26 (89.7)	31.37
		(+-)	1(4.5)	0 (0)	
		(+)	0 (0)	3 (10.3)	
Anti-nRNP/Sm antibodies, n (%)	Before delivery	negative	15 (68.2)	24 (82.8)	31.37
		(+)	1 (4.5)	4 (13.8)	
		(++)	0 (0)	1 (3.4)	
		(+++)	6 (27.3)	0 (0)	
Anti-Ro52 antibodies, n (%)	Before delivery	negative	10 (45.5)	17 (58.6)	31.37
		(+-)	0 (0)	2 (6.9)	
		(+)	3 (13.6)	3 (10.3)	

		(++)	2 (9.1)	2 (6.9)	
		(+++)	7 (31.8)	5 (17.2)	
Anti-SSA antibodies, n (%)	Before delivery	negative	3 (13.6)	17 (58.6)	31.37
		(+-)	4 (18.2)	0 (0)	
		(+)	1 (4.5)	4 (13.8)	
		(++)	2 (9.1)	0 (0)	
		(+++)	12 (54.5)	8 (27.6)	
		**Thyroid Domain (4 variables)**			
free T4 (fT4) (pmol/L)	≤ 13 weeks+6		12.9 (12.4-15.0)	16.1 (12.7-19.1)	58.82
	Before delivery		12.9 (11.9-13.2)	11.9 (10.6-13.0)	29.41
TSH (mIU/L)	≤ 13 weeks+6		1.3 (1.0-2.4)	1.2 (0.2-2.4)	54.90
	Before delivery		1.6 (1.3-2.6)	3.0 (1.7-3.2)	31.37

Data are presented as median value (interquartile range) or number of patients (percentage). In the column named "missing data rate", the underlined values indicates that the missing rate of each variables is more than 30%.

ALT: Alanine Aminotransferase; **AST**: Aspartate Aminotransferase; **ALP**: Alkaline Phosphate; **GGT**: Gamma-Glutamyltransferase; **LDH**: Lactate Dehydrogenase; **CRP**: C-reactive protein; **ESR**: Erythrocyte Sedimentation Rate; **PCT**: Procalcitonin; **Ig**: Immunoglobulin; **ACA**: anti-cardiolipin antibodies; **ANA**: Anti-nuclear antibody; **PT**: Prothrombin Time; **PT-act**: Prothrombin Time Activity; **PT-R**: Prothrombin Time Ratio; **INR**: international normalized ratio; **Fbg**: Fibrinogen; **APTT**: activated partial thromboplastin time; **TT**: thrombin time; **TSH**: thyroid-stimulating hormone;
