# Supplementary figures and images for: Machine Learning Models for Predicting Adverse Pregnancy Outcomes in Pregnant Women with Systemic Lupus Erythematosus
Source: Diagnostics (Basel). 2023 Feb 7;13(4):612. doi: 10.3390/diagnostics13040612 (PMC9955045; doi:10.3390/diagnostics13040612)

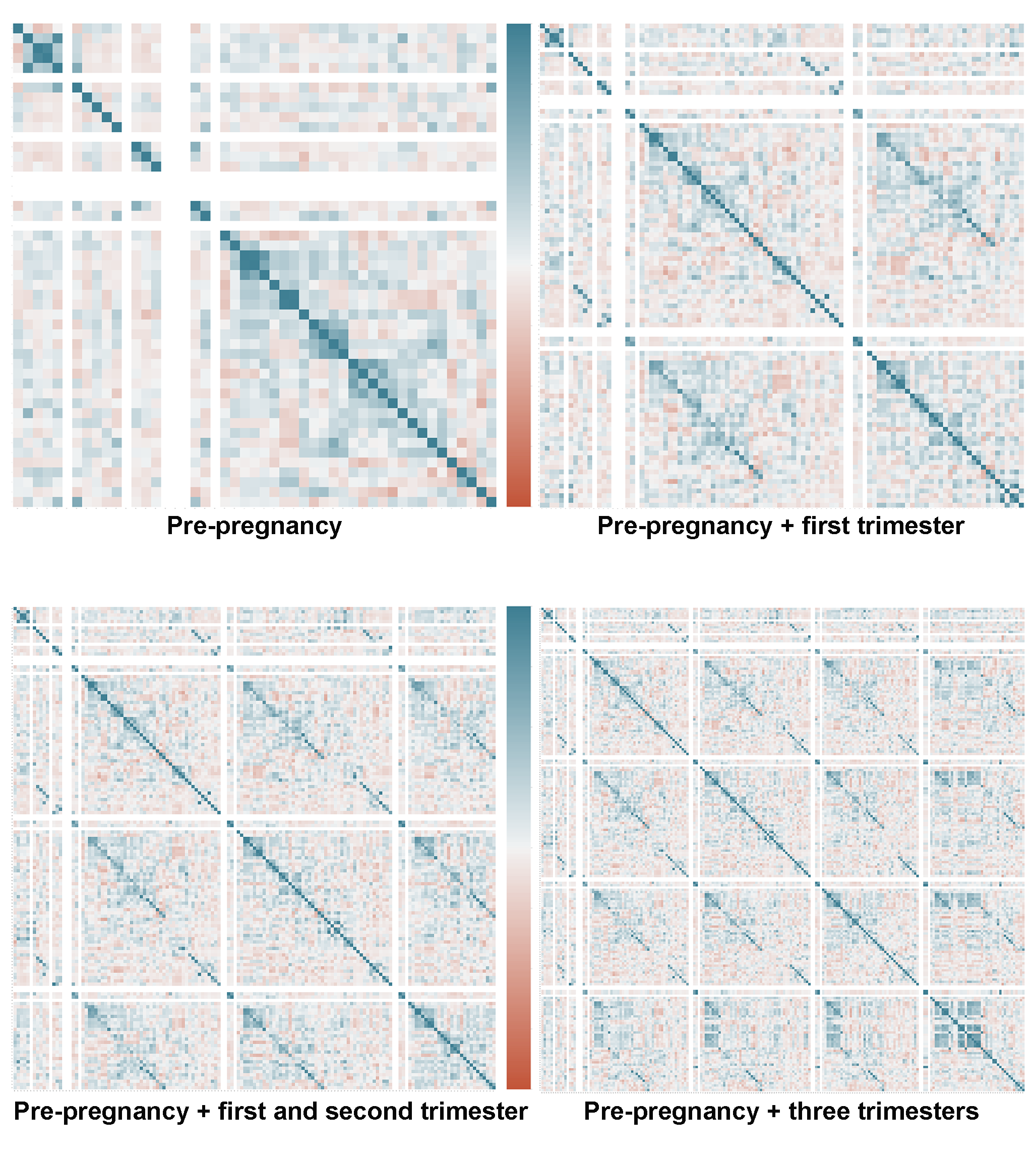

Supplement: Supplementary file 1 [file diagnostics-13-00612-s001.zip › Figure S1. Heat maps generated from four timespans..tif]
